# Profiling miRNAs Involved in Human Oligodendrocyte Precursor Cell Differentiation and Maturation

**DOI:** 10.3390/cells15151343

**Published:** 2026-07-27

**Authors:** Mansoureh Barzegar, Asmita Dhukhwa, Vaidehi Nilesh Patel, Fernanda C. Velasquez, Samarjit Das, Arun H. Patil, Marc K. Halushka, Xitiz Chamling

**Affiliations:** 1 Department of Ophthalmology, Johns Hopkins University School of Medicine, Baltimore, MD 21287, USA; mbarzeg1@jhu.edu (M.B.); asmita.dhukhwa@gmail.com (A.D.); vaidehi.anm@gmail.com (V.N.P.); 2 Department of Anesthesiology and Critical Care Medicine, Johns Hopkins University School of Medicine, Baltimore, MD 21287, USA; fcarrizovelasquez@med.unlp.edu.ar (F.C.V.); sdas11@jhmi.edu (S.D.); 3 Lieber Institute for Brain Development, The Johns Hopkins University, Baltimore, MD 21218, USA; arun.patil@libd.org; 4 Pathology and Laboratory Medicine Institute, Cleveland Clinic, Cleveland, OH 44195, USA; halushm@ccf.org

**Keywords:** microRNAs, human pluripotent stem cells, hPSCs, human oligodendrocyte progenitor cells, hOPC, human oligodendrocytes, human oligodendrocyte differentiation, human oligodendrocyte maturation, microRNA profiling, next generation sequencing

## Abstract

**Highlights:**

**What are the implications of the main findings?**

1. Stage-specific microRNAs identified during human oligodendrocyte differentiation.
2. Novel microRNAs enriched across distinct oligodendrocyte lineage cell developmental stages.
3. microRNA targets implicate AKT and SMAD2/3 signaling pathways.
4. Findings predict microRNA networks regulating human oligodendrocyte maturation.

**Abstract:**

MicroRNAs (miRNAs) are conserved post-transcriptional regulators that play essential roles in cellular development and differentiation. Although miRNAs involved in oligodendrocyte lineage cell (OLLC) differentiation have been extensively studied in rodent models, their functions in human oligodendrocyte (OL) development remain poorly understood. To address this, we used a human embryonic stem cell (hESC) reporter system and an optimized differentiation protocol to generate and isolate well-characterized OLLCs at defined developmental stages. Next-generation sequencing-based miRNA profiling identified stage-specific miRNAs associated with OL lineage specification and maturation. In addition to canonical OL-associated miRNAs, we identified several previously uncharacterized oligodendrocyte progenitor cell (OPC)/oligodendrocyte (OL)-enriched miRNAs, including miR-3943, miR-4286, miR-1296-5p, miR-488-3p, miR-675-5p, and miR-128-3p, as potential novel regulators and molecular markers for human OLLC development. Computational target analysis further identified candidate regulatory genes, including ZNF488, DLX1, CSNK2B, and KCNJ1, and predicted their association with AKT, SMAD2/3, estrogen-receptor, and insulin-signaling pathways. Together, these findings provide a comprehensive stage-specific miRNA resource for human OL lineage development and provide insight into potential miRNA-mediated regulatory networks governing human OL development.

## 1. Introduction

Oligodendrocytes (OLs) are specialized glial cells in the central nervous system (CNS) responsible for forming the myelin sheath that wraps around neuronal axons. Myelin is crucial for the efficient transmission of electrical impulses along neurons. During development, neural progenitor cells (NPCs) in the CNS differentiate into oligodendrocyte progenitor cells (OPCs), which migrate throughout the brain and spinal cord. The OPCs then terminally differentiate into immature OLs. Upon receiving appropriate environmental cues, these immature OLs further mature, wrapping around nearby receptive axons and myelinating them [1]. Myelination of neuronal axons is essential for saltatory conduction of action potential, providing trophic support to the axons, and maintaining their survival and function. Consequently, loss of myelin integrity could lead to severe neurological disorders, such as multiple sclerosis, inherited leukodystrophies, and optic neuritis related to vision loss. To facilitate the repair of damaged myelin and maintain myelin integrity, it is important to understand the molecular mechanisms that regulate OL differentiation and maturation. The process of fate specification at NPC and OPC stages, as well as OL development and maturation, are controlled by several extrinsic and intrinsic factors, which regulate changes at both transcriptional and post-transcriptional levels. MicroRNAs (miRNAs) have been recognized as one of the key post-transcriptional regulators involved in determining the fate of NPCs and their transition into OPC and OL lineage [2,3].

MicroRNAs (miRNAs) are small, noncoding RNA molecules that are trimmed from longer hairpin-loop RNA sequences into functional 19–21 mers by the miRNA processing enzymes DICER1 and DROSHA [4]. miRNAs recognize a complementary sequence in the 3′ untranslated region (UTR) of a protein-coding messenger RNA (mRNA) and inhibit the expression of specific proteins, either by repressing RNA translation or directly promoting the degradation of the associated mRNAs [5]. Each miRNA can target multiple mRNAs, allowing them to modulate numerous pathways, and by regulating molecular pathways, miRNAs have the potential to modulate cell proliferation, differentiation, and lineage specification [6].

miRNAs play a critical role in the development of several CNS cell types, including neurons, astroglia, and oligodendrocyte lineage cells. The aberrant expression of several miRNAs has been implicated in a broad range of neurological disorders, including both demyelination and neurodegenerative diseases, where they may contribute to distinct but sometimes overlapping pathological processes [7]. Oligodendrocyte lineage progression is a dynamic developmental process involving sequential transitions from neural progenitors to OPCs, mature OLs, and myelinating OLs, with each stage characterized by distinct transcriptional and epigenetic programs. Given that miRNAs regulate gene expression in a context- and stage- specific manner, miRNA expression is likely to be temporally regulated to coordinate stage-specific cellular processes during OL development. Consistent with this notion, dynamic regulation of miRNAs in developing OLs has been documented in rodent models [8], particularly by a study in which the deletion of Dicer in OPCs led to delayed OL differentiation and myelination [9]. This and several other studies have identified and confirmed the significance of specific miRNAs, such as miR219 and miR338, in OPC and OL differentiation and maturation [10,11]. However, most miRNA profiling and biochemical studies aimed at understanding the role of miRNAs in OPC and OL development have been performed using murine models. Although human and mouse OPCs and OLs share similarities, several important differences exist [12,13,14], and a comprehensive profiling and characterization of stage-specific miRNA expression during human OL lineage cell (OLLC) development remains lacking.

One of the main challenges in studying these regulatory mechanisms in human OPCs and OLs (hOPCs/OLs) is the limited availability of primary human oligodendrocyte lineage cells, particularly at defined developmental stages. Although primary human tissue provides valuable insights into human OL biology, obtaining sufficient numbers of purified OPCs and OLs representing sequential stages of lineage progression remains challenging due to limited tissue availability, donor variability, and inability to precisely control the developmental stage. Human pluripotent stem cell (hPSC)-derived oligodendrocyte lineage cells (OLLCs) provide a complementary and reproducible model that enables generation and isolation of stage-specific populations under controlled differentiation conditions, making them particularly suitable for studying developmental changes in miRNA expression. However, generating sufficient numbers of well-characterized hPSC-derived OLLCs remains technically challenging. Due in part to this constraint, to our knowledge, only one study profiling global miRNAs in human embryonic stem cell (hESC)-derived OLLCs has been reported to date [15]. Although that study identified several interesting miRNAs, it was limited by the available miRNA profiling technology and the quality of the OPC differentiation protocol at the time [15].

In this study, we used our robust method to generate well-characterized human OPCs and capture miRNAs at seven different stages of OLLC differentiation. We utilized our CRISPR-engineered hESC line, which enables scalable differentiation and enrichment of human OPCs at different developmental stages [16]. The reporter hESC was differentiated into OL lineage and then NPCs, OPCs, and OLs were selectively isolated at different stages of differentiation. We then performed next-generation sequencing (NGS)-based miRNA profiling, which not only identified miRNAs previously shown to be important for mouse OPC differentiation, but also revealed a number of additional miRNAs distinctly enriched at different stages of differentiating human OPC and OL populations.

## 2. Materials and Methods

### 2.1. Human Pluripotent Stem Cells (PSCs) and Culture Conditions

For this study, we used two NIH-approved human embryonic stem cell (hESC) lines, RUES1 and WA09/H9, obtained from WiCell Research Institute (Madison, WI, USA) through a Material Transfer Agreement with them (NIH approval numbers NIHhESC-10-0012 and NIHhESC-10-062, respectively). We also used a hiPSC line (EP1), which was generated in the collaborating laboratory of Dr. Don Zack using IMR90 fibroblast purchased from ATCC (CCL-186, Manassas, VA, USA). This cell line has been used in several previous publications [17,18,19]. hPSCs were maintained on growth factor-reduced Matrigel (354230, Corning, Corning, NY, USA)-coated plates in mTeSR Plus medium (100-0276, Stem Cell Technologies, Vancouver, BC, Canada) at 37 °C under 10% CO_2_ and 5% O_2_ conditions. Cells were dissociated using Accutase (A6964, Sigma-Aldrich, St. Louis, MO, USA) for routine passaging, and 5 μM blebbistatin (B0560, Sigma-Aldrich, St. Louis, MO, USA) was added during the first 24 h after passaging to enhance single-cell survival. For cryopreservation, dissociated cells were resuspended in CryoStore CS10 Freezing Medium (100-1061, STEMCELL Technologies, Vancouver, BC, Canada), frozen at −80 °C overnight using a controlled-rate freezing method and subsequently stored in liquid nitrogen. For thawing, cryovials were rapidly warmed at 37 °C and plated onto Matrigel-coated plates in pre-warmed culture medium. Cells between passages 3–5 after thawing were used for all differentiation experiments. All cell lines were routinely tested for mycoplasma contamination using the MycoAlert detection kit (LT07-318, Lonza, Basel, Switzerland) and qPCR-based analysis, and only contamination-free cells were used for OPC differentiation experiments.

Genomic integrity was further assessed using the KaryoStat+ assay, which enables genome-wide detection of chromosomal abnormalities, including aneuploidies, copy number variations, submicroscopic aberrations, and mosaic events, with a resolution comparable to G-banding karyotyping. The assay detects chromosomal gains and losses larger than approximately 1 Mb, although resolution near telomeric and centromeric regions may be lower. Genomic DNA was isolated using the Genomic DNA Purification Kit and quantified by NanoDrop analysis (Thermo Fisher Scientific, Waltham, MA, USA). A total of 100 ng genomic DNA was processed using the CytoScan HT-CMA 96 array (Thermo Fisher Scientific, Waltham, MA, USA) according to the manufacturer’s protocol. In addition, qPCR-based karyotyping was performed using genomic DNA isolated from bulk cell populations to detect common chromosomal abnormalities and copy number changes associated with pluripotent stem cell culture. Pluripotency and maintenance of the undifferentiated state were assessed prior to differentiation experiments by evaluating the expression of OCT4 using quantitative PCR analysis. Quantitative analysis was performed by calculating relative gene expression normalized to housekeeping controls.

### 2.2. OPC and Oligodendrocyte Differentiation Protocol

The hPSC reporter cell lines (PPM), previously generated by our lab [16,20], were differentiated to OPC and OL following our well-established differentiation protocol [11]. hPSCs were briefly dissociated to single cells and plated on growth factor-reduced Matrigel-coated plates (354230, Corning, Corning, NY, USA) at 200,000 cells/well of a 6-well plate and cultured in mTesr plus (100-0276, Stem Cell Technologies, Vancouver, BC, Canada) at 37 °C, 10% CO_2_/5% O_2_. Neural differentiation and spinal cord patterning was induced by dual SMAD inhibition (SB431542, 10 μM (616464, MilliporeSigma, Burlington, MA, USA) and LDN193189, 250 nM (0559, MilliporeSigma, Burlington, MA, USA) and 100 nM retinoic acid (RA) (R2625, MilliporeSigma, Burlington, MA, USA) two Days after passaging. Differentiating cells were maintained in neural induction media supplemented with RA (100 nM) and smoothened agonist (SAG; 1 mM) (566660, MilliporeSigma, Burlington, MA, USA) from Day 8 to Day 12. Adherent cells were lifted and cultured in low-attachment plates to promote sphere aggregation at Day 12. Spheres were plated into poly-L-ornithine/laminin-coated dishes in OPC/OL differentiating media supplemented with B27 supplement (12587010, Thermo Fisher Scientific, Waltham, MA, USA), N2 supplement (17502048, Thermo Fisher Scientific, Waltham, MA, USA), Platelet-derived growth factor-AA (PDGF-AA) (221-AA-10, R&D Systems, Minneapolis, MN, USA), insulin-like growth factor 1 (IGF-1) (291-G1-200, R&D Systems, Minneapolis, MN, USA), neurotrophin-3 (NT-3) (GF0308, MilliporeSigma, Burlington, MA, USA), Hepatocyte Growth Factor (HGF) (294-HG-025, R&D Systems, Minneapolis, MN, USA), Minocycline (Y0001930, MilliporeSigma, Burlington, MA, USA) 3,3′,5-Triiodo-L-thyronine (T3) (T2877, MilliporeSigma, Burlington, MA, USA), and 3′,5′-Cyclic adenosine monophosphate (cAMP) (D0260, MilliporeSigma, Burlington, MA, USA) at day 30. Cells were fed with this media 2–3 times a week until the end of the differentiation process. 

### 2.3. Flow Cytometry and MACS Purification of the Reporter hOPCs and hOLs

Cells were dissociated into a single cell suspension for flow cytometry analysis and magnetic-activated cell sorting (MACS) purification by incubating them in accutase (A6964, Sigma-Aldrich, St. Louis, MO, USA) for ~45 min. The single cell suspension was subsequently run through a ~70 μm cell strainer (352350, BD Biosciences, San Jose, CA, USA), washed and resuspended in MACS buffer (130-091-221, Miltenyi Biotec, Auburn, CA, USA) for MACS cell sorting or flow analysis. An SH800S Cell Sorter (Sony Biotechnology, San Jose, CA, USA) was used for flow analysis. The number of tdTomato+ or GFP+ cells was measured using live cells. A gate was set up using wild-type hES cells differentiated to Day 75 or Day 95. With a few minor adjustments, MACS purification was performed according to the manufacturer’s instructions. After passing through a cell strainer, cells were resuspended in MACS buffer, to which CD90.2 (THY1.2) (130-121-278, Miltenyi Biotec, Auburn, CA, USA) or O4 MicroBeads (130-094-543, Miltenyi Biotec, Auburn, CA, USA) were added and incubated at room temperature for 15 min for cell binding. The cells were passed through the LS or MS magnetic column (130-042-201, Miltenyi Biotec, Auburn, CA, USA), the columns were washed thrice with MACS buffer, and the cells that were bound to the column were obtained by pushing them through using a syringe. The purity of the tdTomato+ population was increased by passing the collected cells through a new column without additional supplementation of MicroBeads.

### 2.4. miRNA Extraction and Quantification

Total miRNA from three independent biological replicates of a stage-specific cell population were isolated using the miRNeasy Mini/Micro-Kit (217004 and 217084, Qiagen, Hilden, Germany) according to the manufacturer’s instruction. The concentration of the total RNA was measured using Qubit RNA HS assay kit (Q32852, Invitrogen, Thermo Fisher Scientific, Waltham, MA, USA).

### 2.5. miRNA Library Preparation

A miRNA sequencing library was prepared from 10 ng RNA input using QIAseq miRNA library kit (331502, Qiagen, Hilden, Germany) with minor modifications. The quantity and quality of the miRNA library were determined by Qubit DNA HS assay kit (Q32854, Invitrogen, Thermo Fisher Scientific, Waltham, MA, USA) and a fragmented bioanalyzer (JHU Core Facility).

### 2.6. miRNA Profiling and Data Analysis

Following sample collection, RNA extraction, and preparation of miRNA libraries, libraries were sequenced using a full lane of illumina HiSeq (Novogene, Sacramento, CA, USA) with an estimated data output of 110 Gb. After 5% for Phix control, each sample was expected to achieve a 11.6 M paired end read (3.5 Gb data). Sequence trimming, miRNA annotation, and quantification were performed using the Qiagen Geneglobe and miRge3.0 pipeline [21,22]. After trimming the adapter sequences and filtering for the minimum read length of 16, ~80 million trimmed reads out of the total ~300 million reads on average, 40 million reads per sample were achieved. Any sample with a number of reads 5 SD below the average (i.e., one of three Day 8 samples) was excluded from the downstream analysis (Table S1).

### 2.7. qPCR Analysis

Total mRNA from each stage-specific cell population was converted to cDNA using the High-Capacity cDNA Reverse Transcription Kit (4368814, Applied Biosystems, Thermo Fisher Scientific, Waltham, MA, USA). For each qPCR reaction, 1.0 µL of synthesized cDNA was diluted with 1.3 µL of nuclease-free water to prepare a 2.3 µL template mix. Each reaction contained 0.2 µL of custom-designed primers (10 µM) and 2.5 µL of SsoAdvanced Universal SYBR Green Supermix (1725270, Bio-Rad Laboratories, Hercules, CA, USA). A total of 2.7 µL of the primer–supermix mixture was added per well, bringing the final reaction volume to 5.0 µL. Reactions were performed in technical triplicates on a Bio-Rad CFX96 real-time PCR system using SYBR Green detection. Gene expression levels were calculated using the ΔΔCt method, with GAPDH and ACTB used as endogenous controls.

### 2.8. Digital PCR Analysis

Total RNA was extracted using the Qiagen miRNeasy Micro/Mini Kit (217004 and 217084, Qiagen, Hilden, Germany) according to the manufacturer’s instructions. RNA was eluted in 14 µL of nuclease-free water and quantified using nanodrop. Complementary DNA (cDNA) was synthesized from 5 ng of total RNA using miRCURY LNA RT kit (339340, Qiagen, Hilden, Germany). The resulting cDNA was diluted 1:60 in nuclease-free water for digital PCR analysis. Absolute quantification of microRNA was performed using the QIAcuity Digital PCR System (Qiagen, Hilden, Germany) with EvaGreen chemistry (QIAcuity, EG PCR kit, 250111, Qiagen, Hilden, Germany) and LNA-based miRNA assays. Each reaction consisted of 5 µL of 3X EvaGreen Master Mix Qiagen, Hilden, Germany), 5 µL of 10X LNA miRNA PCR assay, 4.75 µL of nuclease-free water and 2 µL of diluted cDNA, for a total reaction volume of 15 µL. Reactions were loaded onto 96-well 8.5k QIAcuity nanoplates (250021, Qiagen, Hilden, Germany) and partitioned according to the manufacturer’s instructions. PCR amplification was performed under standard conditions. Fluorescence imaging of individual partitions was carried out automatically by the instrument. Data were analyzed using QIAcuity Software 2.5.0.1 (QIAGEN, Hilden, Germany) with automatic thresholding. Reactions were performed in technical triplicates. No-template controls (NTCs) were included in each run to assess contamination.

### 2.9. IPA

Ingenuity Pathway Analysis (IPA, Qiagen) was used to predict and analyze the potential downstream targets and biological networks of differentially expressed miRNAs. miRNA lists were uploaded into the IPA software (version 01-23-01), and target prediction was performed based on experimentally validated interactions and high-confidence computational predictions. Core analysis was conducted to identify canonical pathways, upstream regulators, and molecular networks associated with the miRNAs. The resulting interaction networks were visualized within IPA, and key target genes were highlighted for further functional interpretation.

### 2.10. Statistical Analysis

For differential expression analyses of miRNAs, an absolute log2 fold change (|log2FC|) > 1 and a false discovery rate (FDR)-adjusted *p* value < 0.01 were considered significantly differentially expressed, unless otherwise specified. For qPCR analysis, statistical analyses were performed using GraphPad Prism (version 10.6.1). Data are presented as mean ± standard error of the mean (S.E.M.) from three biological replicates. Comparisons between multiple groups were conducted using one-way analysis of variance (ANOVA) followed by appropriate post hoc tests. A two-tailed *p* value < 0.05 was considered statistically significant.

## 3. Results

### 3.1. miRNA Isolation from hESC-Derived Cells at Different Stages of Oligodendrocyte Differentiation

**Figure 1:**
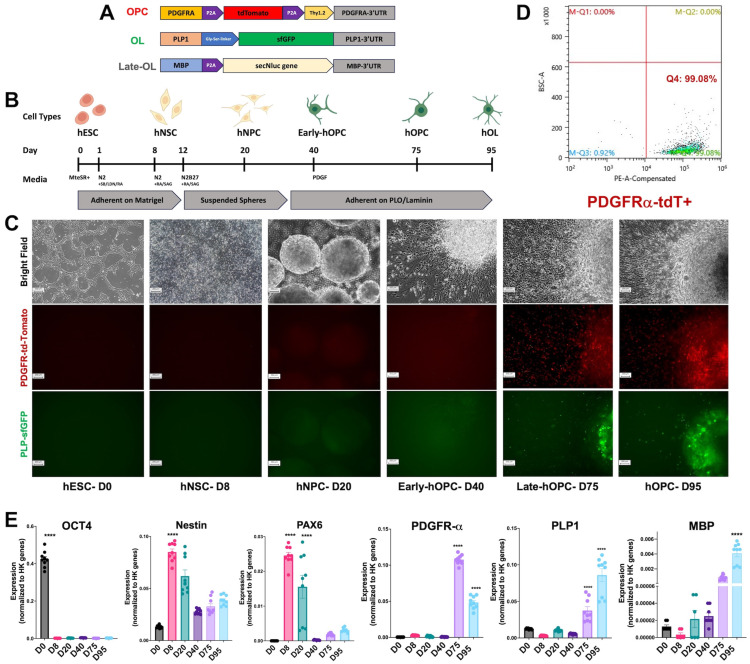
miRNA isolation from a reporter hESC-derived cells at different stages of oligodendrocyte differentiation. (**A**) Schematic of the CRISPR-Cas9-engineered hESC reporter cell line (PPM). (**B**) Timeline showing differentiation and maturation of hOPCs and hOLs derived from PPM cell line. (**C**) Representative images of stage-specific cell population during the differentiation process. (**D**) An example of flow cytometry analysis showing 99% tdTomato+ hOPCs following purification using Thy1.2 antibody conjugated magnetic microbeads. (**E**) qPCR confirming the expression of stage-specific markers in each cell population. D95 sample represents the O4-enriched cells. Three clonal lines were used as biological replicates. Statistical significance was calculated using one-way ANOVA, **** *p* < 0.0001. Data represents mean values ± S.E.M.

To comprehensively profile miRNA expression at various stages of human OLLC development, we utilized a hESC (RUES1) reporter system that we developed in our laboratory [16,20] (Figure 1A). As previously reported [16], this reporter cell line, referred to as PPM (PDGFRα-PLP1-MBP reporter) hereafter, consists of three reporters: (1) PDGFRα-P2A-tdTomato-P2A-Thy1.2, where endogenous *PDGFRα* expression leads to the production of tdTomato fluorescent marker and cell-surface protein, Thy1.2. The tdTomato protein localizes to the cytoplasm whereas Thy1.2 localizes to the cell surface, enabling the immunopurification of PDGFRα-expressing cells using anti-Thy1.2 antibody; (2) PLP1-sfGFP, in which expression of sfGFP is driven by an early OL marker *PLP1*; and (3) MBP-P2A-secNLuc, where NanoLuc protein is secreted into the cell culture media, enabling serial sampling of the media for quantitative analysis of *MBP* expression [16].

We differentiated three hESC clonal lines of this reporter system into hOPC/OL following our established protocol [16,23] and collected cell populations at defined stages: stem cells (SC) at Day 0, neural stem cells (NES) at Day 8, neural progenitor cells (NPC) at Day 20, and early OPCs at Day 40 (Figure 1B,C). At Day 75, post-mitotic PDGFRα-tdTomato+ OPCs were purified using magnetic-activated cell sorting (MACS) with Thy1.2 antibody-conjugated microbeads. Using this MACS method, we regularly achieve >95% tdTomato+ OPC population as confirmed with flow cytometry analysis (Figure 1D). The corresponding flow-through (FT) fraction represented the non-OPC population, which, based on our previous studies, consists primarily of astroglia cells with a small fraction of neurons and neural precursor cells [16,24]. At Day 95 of differentiation, oligodendrocytes lineage cells were enriched using O4 microbeads, which recognizes the sulfatide antigen expressed on pre-myelinating and early OLs. Although it is possible to sort OLs based on PLP1-GFP expression using fluorescence-activated cell sorting (FACS), we found that FACS substantially reduced cell viability and compromised RNA quality. Therefore, MACS-based enrichment was used to collect O4+ oligodendrocytes. The identity of all developmental populations, including O4-enriched D95 cells, was further verified by qPCR analysis of stage-specific markers, including *OCT4* for SC, NES and *PAX6* for NPCs, *PDGFRα* for early OPCs, and *MBP* and *PLP1* for OLs (Figure 1E).

### 3.2. Next-Generation Sequencing-Based miRNA Profiling

miRNA profiling has traditionally been performed using microarray platforms, the NanoString nCounter system, or quantitative real-time PCR, each of which has limitations that impact their ability to provide a broad and accurate quantification of miRNAs [25]. Recent advancements in next-generation sequencing (NGS) methods offer a more advantageous approach to miRNA profiling compared to these other platforms, as the NGS-based method is not limited by predefined features, probe design, or array background [26]. NGS is not only suitable for confirming the known miRNAs, similar to qRT-PCR and microarray platforms, but it can also detect novel miRNAs. Therefore, we opted to use the Qiagen miRNA library prep and Illumina Hiseq for this miRNA profiling study (Figure S1).

**Figure 2:**
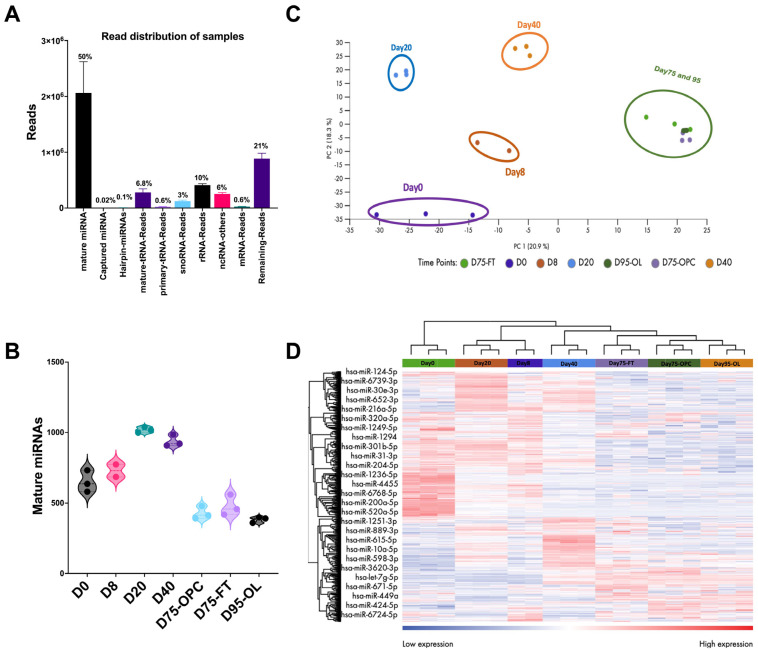
The NGS-based approach efficiently captured mature miRNA. (**A**) Distribution of various small RNA species captured across samples. The Y-axis represents the number of sequencing reads, and X-axis indicates different types of small RNAs detected. The percentage of filtered reads corresponding to each small RNA type is displayed above each bar. (**B**) Distribution of the number of mature miRNAs captured at different time points during the differentiation process. (**C**) Principal Component Analysis (PCA) of miRNA expression profiles across distinct differentiation time points. Samples are represented as points, and their clustering reflects similarities or differences in miRNA expression patterns during OL differentiation. (**D**) Heatmap of differentially expressed miRNAs during different stages of OL development. Rows represent individual miRNAs, and columns correspond to differentiation time points. Expression levels are visualized using a color scale, with statistical significance set at false discovery rate (FDR) *p* value < 0.01.

This NGS-based approach efficiently captured mature miRNA, with approximately 50% of the reads (~40 million of the ~80 million trimmed reads from the 20 runs) mapping to mature human miRNA in miRbase (Figure 2A, Tables S1 and S2). An average of 657 mature miRNAs were identified across each cell population, with the median range between 391 and 1017 miRNAs (Figure 2B). The highest number of miRNAs were captured in the D20 NPC population, while the O4+ OLs had the least (Figure 2B). Principle component analysis (PCA) showed clear separation of clusters corresponding with different time points, highlighting that temporal changes in miRNA expression are strongly associated with the differentiation process. When compared to the SC stage, the most pronounced separation was observed in more mature cells at Day 75 and Day 95 (D75 OPCs, D75 non-OPC (flow-through; FT), and D95 O4+ OL). Among the more mature populations, OPC (Day 75) and OL (Day 95) clusters were closer to each other than the non-OPC population (D75-FT) (Figure 2C). The heatmap of differentiation stage-specific differentially expressed miRNAs further underscores these findings, revealing approximately 900 miRNAs that are differently expressed during the differentiation process (Figure 2D). The majority of miRNA that were highly expressed during the SC stage showed a progressive reduction as the cell matured, and vice versa (Figure 2D). Overall, these results show that miRNA expression changes more markedly during OLLC differentiation than between OPC/OL and non-OPC lineage cells sampled at similar time-points. Our NGS-based miRNA profiling therefore provides a comprehensive overview of miRNA dynamics during human OL lineage development, revealing key miRNAs that potentially regulate maturation and lineage specification at various stages.

### 3.3. Stage-Specific Enrichment of Previously Characterized miRNAs During OL Development

**Figure 3:**
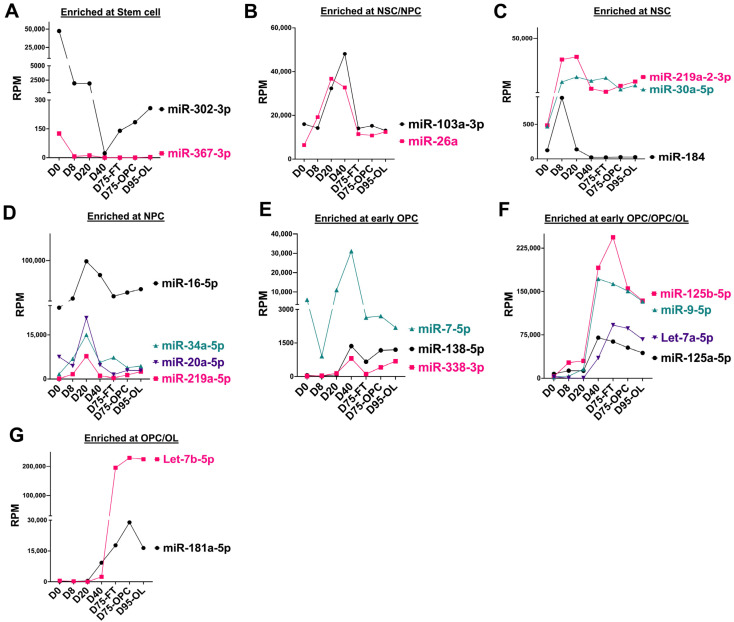
Stage-specific enrichment of previously characterized miRNAs during OL development. Each graph illustrates the expression patterns of miRNAs enriched at specific developmental stages: (**A**) Day 0 representing the pluripotent stem cell stage. (**B**) Neural stem cells (NSCs) and neural progenitor cells (NPCs) at Days 8 and 20. (**C**) NSCs at Day 8. (**D**) NPCs at Day 20. (**E**) Early OPCs at Day 40. (**F**) Early OPCs, mature OPCs, and OLs. (**G**) Mature OPCs and OLs at Days 75 and 95. Each data point represents the average reads per million (RPM) from three biological replicates.

As a validation of our miRNA data, we assessed the presence of miRNAs known to be upregulated in various cell types. As expected, miR-302b-3p, which is known to be upregulated in human pluripotent stem cells (hPSCs) [27,28] and has been reported to promote reprograming of human somatic cells to hPSCs [29], was highly enriched in the hESCs but not in other populations (Figure 3A). miR-26b-5p [30] and miR-103a-3p [31], which have been reported to initiate ESC to NPC differentiation and whose continued enrichment leads to neural differentiation in murine ESC, were enriched in our D20 NPCs and D40 progenitor cells but showed a reduced expression in the OPC and OL population (Figure 3B).

miR-219 family members, namely miR-219a-2-3p and miR-219a-5p, were enriched in OPCs and OLs compared to the non-OPC population; however, their highest expression was detected in the D20 NPCs when compared to other time-points (Figure 3C,D). Similar to previous reports [32,33], this data suggests that continued expression of miR-219a-5p and miR-219a-2-3p is crucial to NPC differentiation and important for OL lineage specification. Similarly, miR-138, and miR-338-3p, which have been implicated in OPC differentiation and OL maturation [9,32,33,34], were enriched in D40 precursors, OPCs and Ols, but their expression was significantly reduced in the non-OPC population (Figure 3E).

We also compiled a list of approximately 150 miRNAs previously reported to be involved in OPC and OL differentiation and maturation (Table S3) and assessed their expression in our dataset. Since most of these reports are based on rodent models, we observed only limited overlap with our data. However, stage-specific enrichment of several miRNAs was conserved between the previous reports and our dataset. Notably, enriched expression of miR-7-5p, miR-16-5p, miR-20a-5p, and miR-34a-5p in NPCs and early OPC was also observed in our dataset, suggesting their involvement in the initiation of OL differentiation [35,36] (Figure 3D,E). Several other miRNAs, including miR-9-5p [37,38], miR-125b-5p, miR-125a-5p [39], and let-7a-5p, also showed increased expression in the hOPC (Day 75 population) in our dataset (Figure 3F). Notably, let-7b-5p and miR-181a-5p [9,40,41] showed a significantly increased expression at the OPC stage, which remained elevated at a later time point (OL, Day 95) (Figure 3G). These data validate that our reporter hESC-derived cells and the miRNA profiling method can effectively capture mature miRNAs involved in OLLC development but also highlight a significant difference in the miRNAs that are involved in human vs. mouse OLLC differentiation.

Nonetheless, it is worth noting that several miRNAs previously implicated in human ESC differentiation to OL lineage [15] did not show expression in the expected populations in our data. For example, miR-184, which is reported to be specific to OL [15], was enriched only in the NPCs but significantly reduced in OPCs and OLs (Figure 3C); miR-367 that is reported to be enriched in hESC, NPCs and OPCs [27,42] had a higher expression in hESCs compared to NPCs but it was not detected in the other populations (Figure 3A); and miR-663, miR-1225-5p, miR-638 that were previously reported to be enriched in hESC-derived OL [15] were surprisingly not detected in any cell population in our dataset.

### 3.4. Identification of Differentiation Stage-Specific Enriched miRNAs

**Figure 4:**
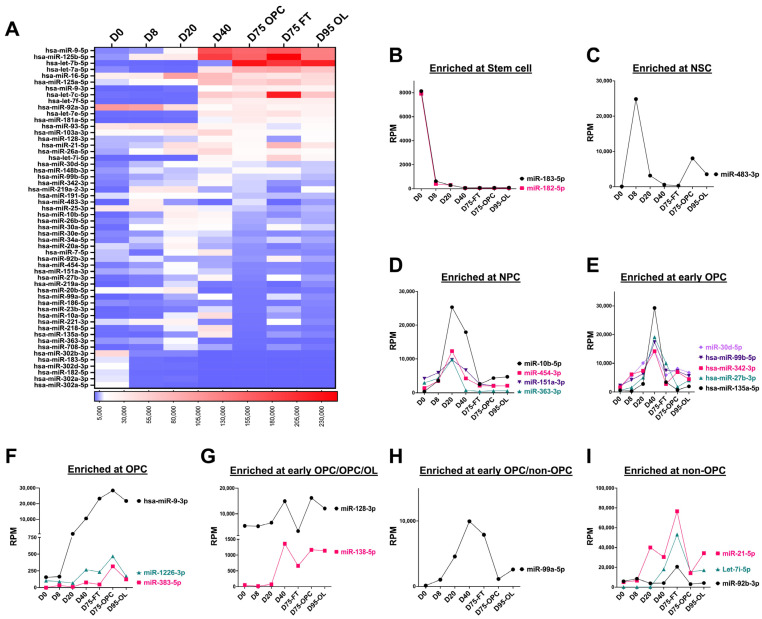
Variance analysis reveals stage-specific enrichment of miRNAs during OL differentiation. (**A**) Heatmap showing the top-variance miRNAs across OPC to OL differentiation. (**B**–**I**) Graphs illustrating the stage-specific enrichment of miRNAs uniquely identified in our dataset. (**B**) Expression pattern of miRNAs enriched in pluripotent stem cell population (Day 0); (**C**) NSC (Day 8); (**D**) NPC (Day 20); (**E**) early OPC (Day 40); (**F**) OPC (Day 75); (**G**) Early OPC/OPC/OL (Day 40, 75 and 95); (**H**) early OPC and non-OPC (Day 40 and flow-through from Day 75); and (**I**) only non-OPC (flow-through from Day 75). Each data point represents the average normalized RPM from three biological replicates.

To identify miRNAs with marked differential expression across different OLLC differentiation stages, we calculated the variance in miRNA expression across various time points and selected the 50 miRNAs with the highest variance (top second percentile) across different time-points (Figure 4A). This analysis revealed several known miRNAs, as well as a few novel miRNAs, that are enriched in a stage-specific manner. For example, in addition to miR-302b-3p and miR-302a-3p, which were previously reported, we found a highly specific and significant enrichment of miR-183-5p and miR-182-5p at the stem cell (SC, Day 0) stage (Figure 3A and Figure 4B). miR-483-3p was specifically enriched at the neural stem cell (NSC, Day 8) stage (Figure 4C), while the upregulation of miR-454-3p, miR-363-3p, miR-151a-3p, and miR-10b-5p was highly specific to the neural progenitor cell (NPC, Day 20) stage (Figure 4D). Additionally, miR-135a-5p, miR-27b-3p, miR-342-3p, miR-99b-5p, and miR-30d-5p exhibited specific enrichment during the pre-OPC stage (Day 40) (Figure 4E). We also identified two miRNAs, miR-1226-3p and miR-383-5p, specifically enriched in the OPC (D75) population (Figure 4F). A drastic increase in the expression of miR-9-3p was observed during differentiation, with OPC-specific further enrichment (Figure 4F).

The variance analysis also highlighted two miRNAs, miR-138-5p and miR-128-3p, which are enriched in the early-OPC population (Day 40), with this enrichment persisting in the OPC and OL populations (Figure 4G). When compared to the OPC/OL population, the expression of both miRNAs, particularly that of miR-128, was significantly reduced in the non-OPC populations, suggesting their importance in maturation of neural stem cells into OLLCs. On the other hand, miR-99a-5p was upregulated in early OPC and non-OPC (Day 75 FT) populations (Figure 4H), while miR-92b-3p, let-7i-5p, and miR-21-5p were enriched only in the non-OPC population (Figure 4I). Since our previous study demonstrated that the flow-through after OPC immune-purification primarily comprises astrocyte cells [16], these data suggest a potential role for these miRNAs in the specification of astrocytes from NPCs. Overall, this analysis helped generate a list of candidate miRNA markers specific to different stages of human OLLC differentiation.

### 3.5. Validation of Promising Candidate miRNAs in Multiple Cell Lines

**Figure 5:**
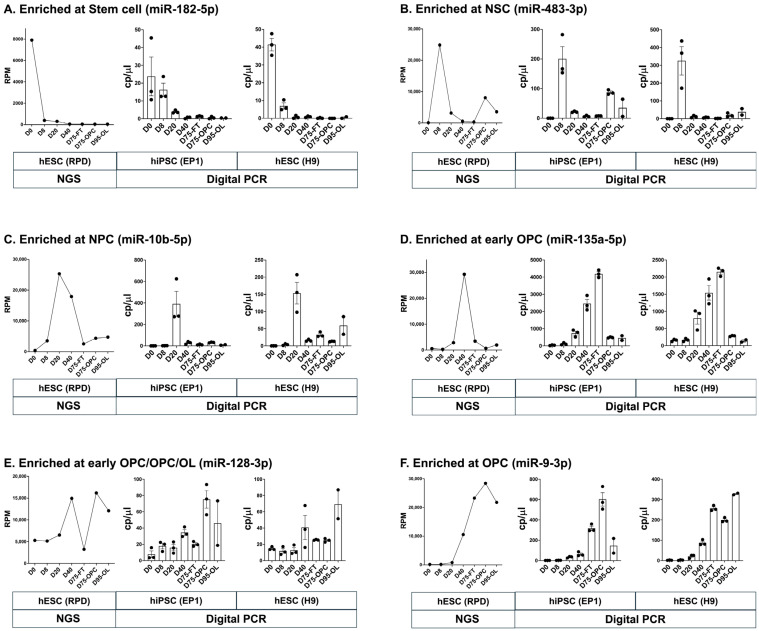
Digital PCR analysis validates the enrichment of selected miRNAs at different stages of OL differentiation. Graphs showing the similarity between miRNA expression pattern captured by NGS and absolute copy number (cp/µL) counts measured by dPCR. Selected miRNAs enriched at distinct stages of lineage progression were validated using dPCR in hESC (H9)- and hiPSC (EP1)-derived OLLCs. NGS-based expression patterns and digital PCR measurements are shown for: (**A**) miR-182-5p, enriched in stem cells (Day 0); (**B**) miR-483-3p, enriched in neural stem cells (Day 8); (**C**) miR-10b-5p, enriched in neural progenitor cells (Day 20); (**D**) miR-135a-5p, enriched in early oligodendrocyte precursor cells (OPC) (Day 40); (**E**) miR-128-3p, enriched across early OPC/OPC/OL stages; and (**F**) miR-9-3p, enriched in OPCs (Day 75). Each data point in the digital PCR plots represents three technical replicates.

To validate the stage-specific enrichment of the candidate miRNAs identified by miRNA profiling, we performed digital PCR on OLLCs generated from two additional hPSC lines: a hESC line (H9) and an iPSC line (EP1). dPCR-based absolute quantification confirmed the distinct temporal patterns of miRNA expression corresponding to specific OLLC stages. Consistent with the profiling data, miR-182-5p was enriched in stem cells; miR-483-3p at NSC-D8; miR-10b-5p at NPC-D20; miR-135a-5p at early OPC-D40; miR-9-3p at OPC stages; and miR-128-3p in both OPCs and oligodendrocytes (Figure 5A–F and Figure S2A–F). Although expression differences for a couple of miRNAs were observed between OPC/OL and non-OPC populations, these likely reflect variation in OPC purity and maturation between samples (Figure S3A,B). Overall, the candidate miRNA expression patterns were largely consistent across hESC- and hiPSC-derived OLLCs, supporting the reproducibility of these stage-specific miRNA signatures for future studies.

### 3.6. miRNAs That Are Differentially Expressed in Human OPC and OLs Populations

**Figure 6:**
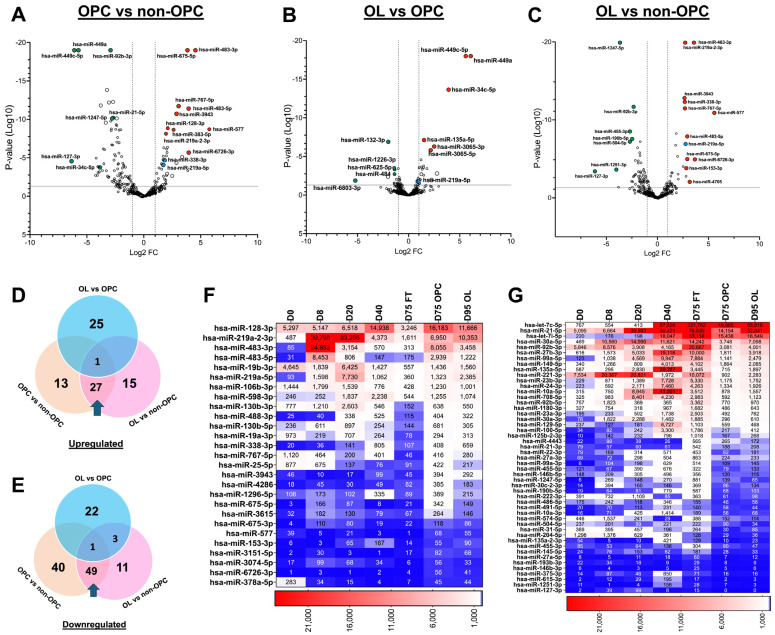
OPC- and OL-specific miRNA enrichment analysis. Volcano plot showing differentially expressed miRNAs between: (**A**) OPC and non-OPC, (**B**) OL and OPC, and (**C**) OL and non-OPC populations. A log2 fold change > 1 or <−1 with FDR *p*-value < 0.01 is considered significant. Venn diagrams illustrating (**D**) upregulated and (**E**) downregulated miRNAs across the three comparisons. miR-219-5p is identified as the commonly upregulated miRNA, while miR-1247-5p is the commonly downregulated miRNA across all comparisons. Heatmap of (**F**) 27 upregulated and (**G**) 49 downregulated miRNAs from the comparisons of OPCs vs. non-OPCs and OLs vs. non-OPCs populations highlights miRNAs specifically enriched in OPC/OL populations.

Since OPC-to-OL differentiation is inhibited in MS and other demyelinating diseases, we were particularly interested in identifying miRNAs that are potentially involved in OPC formation and their maturation into myelinating OLs. Therefore, we performed differential expression analysis among three mature cell populations: OL vs. OPC, OPC vs. non-OPCs, and OLs vs. non-OPC samples (Figure 6A–C). This analysis revealed 52 (26 up/26 down), 129 (40 up/89 down), and 106 (42 up/64 down) differentially expressed miRNAs (log2 FC > 1 or <−1 and FDR *p*-value < 0.01) in the OL vs. OPC, OPC vs. non-OPC, and OLs vs. non-OPC datasets respectively (Figure 6D,E). Interestingly, only miR-219-5p was consistently upregulated and only miR-1247-5p was consistently downregulated across all three datasets, highlighting the potential roles of miR-219-5p upregulation and miR-1247-5p downregulation in OPC lineage selection and OL maturation.

Among the miRNAs upregulated in Day 95-OL when compared to Day 75-OPC are miR-449c-5p, miR-449a, and miR-34c-5p. Additional miRNAs such as miR-135a-5p, miR-3065-3p, miR-3065-5p, and miR-221-5p were also upregulated in OL when compared to OPC, although to a lesser extent (Figure 6B). Interestingly, many miRNAs enriched in the OL population, including miR-449c-5p, miR-449a, and miR-34c-5p, were also enriched in the non-OPC (flow-through; FT) population when comparing OPCs vs. non-OPCs (Figure 6A). This observed overlap in miRNA enrichment could result from inadvertent inclusion of non-specific cells in a sample. For example, during MACS purification, which selectively isolates PDGFRα-tdTomato-expressing OPCs, a small population of mature OLs may remain in the flow-through, leading to a higher expression of OL-associated miRNAs in the non-OPC samples. A similar inadvertent inclusion of a non-specific cell population could occur in the D95 OL population isolated using O4 antibodies. Although this method selects cells significantly enriched for OL markers (Figure 1E), it may also contain a small fraction of astrocytes, another major cell type in our differentiating culture system [16]. Since astrocytes make up a significant proportion of the non-OPC FT sample, miRNAs commonly enriched in both OLs and non-OPC may instead be astrocyte enriched.

Therefore, to identify miRNAs specifically associated with OPC/OL populations, we focused on candidates enriched in both OPCs and OLs when compared to non-OPC populations. In this analysis, 27 miRNAs were commonly upregulated, and 49 miRNAs downregulated in both OPC vs. non-OPC and OL vs. non-OPC datasets (Figure 6D,E). Among the miRNAs enriched in OPCs and OLs were miR-219a-5p and miR-338-3p, two widely studied miRNAs known for their roles in OL differentiation and myelination, further validating our method and our findings. Similarly, miR-128 expression is upregulated at Day 40 pre-OPCs and remains enriched in OPCs and OLs but not in non-OPC population (Figure 6F), suggesting its role in OPC/OL differentiation. In line with these findings, miR-128-3p was recently reported to reverse fibrinogen-mediated inhibition of OPC differentiation and remyelination following cerebral ischemia [43].

In addition, we identified several miRNAs that have not been reported or extensively studied in OPC/OL biology. Notably, at least eight miRNAs (miR-3943, miR-1296-5p, miR-4286, miR-3615, miR-675-5p, miR-3151-5p, 6726-3p, and miR-577) were specifically enriched during the OPC and OL stages, suggesting their important role in OLLC differentiation (Figure 6F). Conversely, among the downregulated miRNAs, miR-92b-5p/3p, miR23a-3p, miR4443, and miR-21-3p were among the candidates specifically enriched in the non-OPC FT population (Figure 6G). The stage-specific enrichment of these miRNAs suggests their involvement in OPC lineage maintenance and their eventual differentiation into oligodendrocytes. These findings also emphasize the dynamic regulation of miRNAs during differentiation and highlight stage-specific candidates that are likely to play crucial roles in OLLC fate determination.

### 3.7. Potential Biological Pathways and Molecular Networks Regulated by Differentially Expressed miRNAs

**Figure 7:**
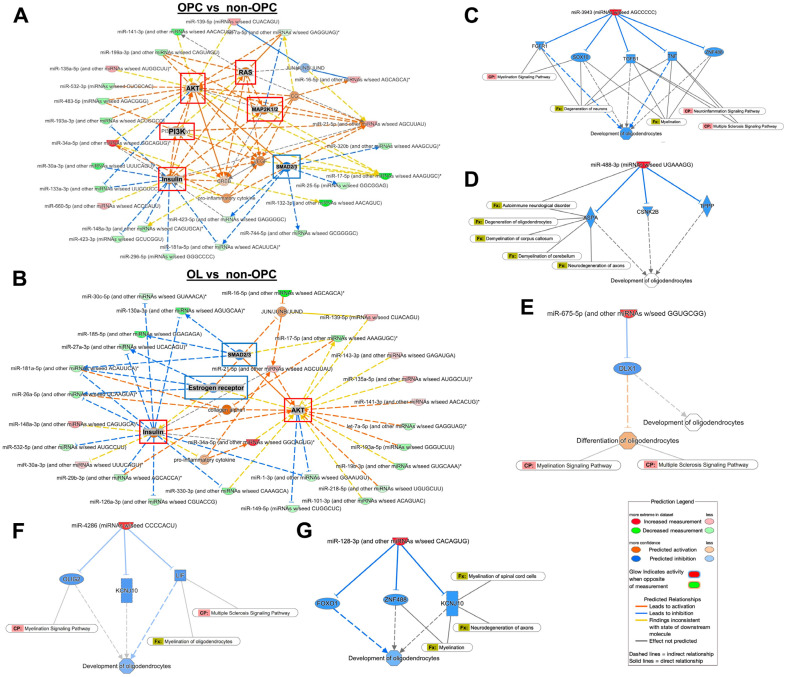
Ingenuity Pathway Analysis (IPA) of enriched miRNAs. (**A**,**B**) IPA was performed on differentially expressed (DE) miRNAs identified in two comparisons: (**A**) OPC vs. non-OPC and (**B**) OL vs. non-OPC. (**A**) DE miRNAs in OPCs relative to non-OPCs are associated with the predicted activation (red lines) of AKT, PI3K, RAS, MAP2K1/2, and insulin-signaling pathways, alongside the inhibition (blue lines) of the SMAD2/3 pathway. An asterisk (*) in the networks indicates an IPA miRNA family node, representing the indicated miRNA together with other miRNAs sharing the same seed sequence. (**B**) The DE miRNAs in OL vs. non-OPC comparisons are associated with predicted activation (red lines) of AKT, and inhibition (blue lines) of the estrogen receptor and SMAD2/3 pathways (**D**,**E**). Predicted targets of OPC/OL-enriched miRNAs through which they potentially affect OL development and function: (**C**) miR-3943, (**D**) miR-488-3p, (**E**) miR-675-5p, (**F**) miR-4286, (**G**) miR-128-3p.

To investigate potential biological pathways and molecular networks associated with differentially expressed miRNAs, we performed Ingenuity Pathway Analysis (IPA) for each comparison group: OPC vs. non-OPC, OL vs. non-OPC, and OL vs. OPC (Figure 7A,B and Figure S4). IPA predicted that a substantial proportion of OPC/OL-enriched miRNAs, as well as several miRNAs downregulated in comparison to non-OPCs (FT), were associated with a predicted activation of AKT, PI3K, and the insulin-signaling pathway, and with the inhibition of the SMAD2/3 pathway. Additionally, inhibition of the estrogen receptor pathway was predicted in the OL vs. non-OPC comparison (Figure 7B), which aligns with previous studies reporting that the pharmacological modulation of this pathway enhances OL maturation [44,45].

In this IPA-based predictive model, multiple miRNAs that were reduced in OPCs and enriched in OLs, including miR-22-3p, miR-135a-5p, miR-30c-5p, and miR-148a-3p, were associated with predicted inhibition of ERK1/2 and AKT signaling (Figure S4A). Similar to OPC vs. non-OPC, IPA also predicted the downregulation of SMAD2/3 signaling, suggesting that changes in these pathways may be associated with a transition from OPC to mature OLs. These predictions are consistent with previous studies demonstrating roles for AKT and SMAD2/3 signaling in OL lineage progression. For example, AKT signaling promotes OL differentiation by phosphorylating FoxO1 and subsequent regulation of Sox10 expression, a key transcription factor in OL development [46]. Similarly, SMAD2/3, as downstream effectors of TGF-β signaling, have been implicated in multiple aspects of OL biology. During early stages, SMAD2/3 activation can influence lineage commitment [47,48], while at later stages it may regulate extracellular matrix remodeling, OL maturation, and interaction with astrocytes and microglia in the myelination environment [49,50].

Next, we mapped the OPC/OL-enriched miRNAs, namely miR-3943, miR-4286, miR-1296-5p, miR-488-3p, miR-1226-3p, miR-675-5p, miR-483-5p and miR-128-3p, to their predicted downstream target molecules (Figure 7C–G and Figure S4B–G). This analysis identified several candidate target genes potentially associated with these miRNA expression patterns, including *ZNF488, CNSK2B, OLIG2* and *SOX10*. Additional predicted miRNA–target relationships included *ASPA* and *TPPP* for miR-488, *KCNJ10* and *FOXO1* for miR128-3p, LIF for miR-4286, and *DLX1* for miR-675-5p (Figure 7 and Figure S4). While the majority of these genes have an established role in OL biology, their functional regulation by specific miRNAs, particularly within the human cell system, remains to be experimentally validated. Therefore, these predicted miRNA–target interactions represent potential regulatory candidates for future mechanistic studies and may provide insights into key molecular intermediates through which miRNAs could influence OL development and maturation [51,52,53,54,55,56].

## 4. Discussion

Since the discovery of miRNA in *C. elegans* in 1993 [57] and the subsequent demonstration that they are evolutionarily conserved across species, it has become clear that they control temporal transitions during development across animal phylogenies [58]. This discovery has sparked an explosion of research focused on miRNAs, leading to the recognition of their crucial roles in development and organogenesis, as well as in the pathogenesis of numerous diseases, including cancer, cardiovascular disease, and neurodegenerative disorders [59]. An increasing number of studies have investigated the role of miRNAs in the development and function of CNS cell types, including OPCs and OLs. These studies show that several miRNAs are differentially expressed at distinct stages of OL differentiation, from progenitor cells to mature oligodendrocytes. Notably, miR-219 and miR-338 have been identified as key regulators of OL differentiation by targeting inhibitors of OL maturation and myelination pathways [9,10]. miR-219 is particularly important for both early and late stages of differentiation, while miR-338 functions primarily during the maturation phase, often acting synergistically with miR-219. Furthermore, overexpression of miR-219 has been reported to promote remyelination in demyelination models [9,10]. miRNA expression profiling during OL development has revealed additional miRNAs, including miR-23a, miR-27a, and miR-29a, which appear to fine-tune the timing of OL maturation and myelin production [60,61]. Other miRNAs such as miR-124 and miR-9 have also been implicated in maintaining the balance between OPC proliferation and differentiation by regulating transcription factors and signaling pathways essential for OL lineage progression [8,62,63]. In addition to development, changes in the miRNA profiles of neurons, microglia, OPCs, and OLs in response to disease and disease-associated stressors have also been reported [64,65]. For example, altered levels of miR-146a and miR-155 have been associated with multiple sclerosis (MS), indicating their potential roles in inflammatory responses affecting OLs [36,66].

However, it is important to note that the majority of studies examining miRNA roles in OL lineage cell (OLLC) development have been conducted using rodent models. Given the fundamental molecular differences between human and rodent OPCs and OLs [12,13,14], there is a clear need to investigate the miRNAs that regulate human OL development, lineage specification, and function. This is particularly important for improved disease modeling and for supporting therapeutic discovery in demyelinating diseases. To our knowledge, only one published study has profiled global miRNA expression in human ESC-derived OLLCs [15]. However, this study did not detect miR-219 or miR-338, two of the most consistently implicated miRNAs in OL differentiation and known to be highly enriched in primary human OPCs and OLs [10,15], in any of their OLLC populations. Moreover, no follow-up studies have validated or further characterized the OPC/OL-associated miRNAs identified in that study. Given that the differentiation methods used at the time were less robust, it is likely that several false positives were discovered in this study. Additionally, the microarray and NanoString nCounter platforms used in many prior miRNA profiling studies, including this study, are limited by their reliance on predefined probe sets, which restricts the ability to detect novel miRNAs or low-abundance RNA molecules [25]. In contrast, NGS-based methods offer a more comprehensive and dynamic approach to miRNA profiling, with higher sensitivity and lower false-positive rates compared to microarrays [67,68]. For this reason, we employed our human ESC reporter system [16,20] that enables us to track and isolate well-characterized hOPCs and OLs, combined with an improved hOPC/OL differentiation protocol, to collect OLLCs at various developmental stages. We then performed miRNA profiling using an NGS-based approach (Qiagen miRNA library prep and Illumina HiSeq), which allows for a more detailed and accurate expression analysis.

Comprehensive miRNA profiling during the differentiation of hOLLCs using our reporter cell line identified several previously known as well as novel miRNAs with potential roles in OLLC differentiation. We observed the enrichment of well-characterized miRNAs at specific stages, including miR-302b-3p at the stem cell stage [27,28]; miR-26b-5p [30] and miR-103a-3p in NPCs [31]; and miR-16-5p, miR-20a-5p, and miR-34a-5p in early OPCs [35,36]. miR-181a-5p, which has been implicated in both neural and oligodendrocyte development as well as in the regulation of myelination-related genes [40,41], showed moderate expression during early differentiation, with levels gradually increasing and peaking at Days 75 and 95. Importantly, the expression of stage-specific marker genes (Figure 3), together with the OPC- and OL-specific enrichment of miR-219a-5p, miR-338-3p, and miR-138 (both of which are known to cooperate with miR-219 in regulating OPC and OL differentiation [9,32,33,34]) further confirmed the quality of our differentiated cell populations and the reliability of our dataset. Among the lesser-studied miRNAs, we observed distinct stage-specific enrichment patterns: miR-182 in pluripotent stem cells (PSCs); miR-483-3p in neural stem cells NSCs (Day 8); miR-454-3p, miR-363-3p, miR-151a-3p, and miR-10a-5p in NPCs (Day 20); and miR-135a-5p, miR-27b-3p, and miR-342-3p in pre-OPCs (Day 40). We confirmed the presence of several of these miRNAs across multiple hESC/iPSC lines using dPCR and provide evidence that they could serve as useful molecular markers for identifying human CNS cell types in future studies.

We also identified several miRNAs specifically enriched in OPCs, including miR-3943, miR-1296-5p, miR-4286, miR-3615, miR-675-5p, miR-3151-5p, miR-6726-3p, and miR-577. Conversely, miR-92b-5p/3p, miR-23a-3p, miR-4443, and miR-21-3p were specifically upregulated in the non-OPC (FT) population. Interestingly, several miRNAs previously reported to be involved in human ESC differentiation toward the OL lineage [15] did not show the expected expression patterns in our dataset. For example, miR-184, previously described as OL-specific, was enriched only in the NPC population and significantly downregulated in both OPCs and OLs (Figure 3B). miR-367, reported to be expressed in ESCs, NPCs, and OPCs [27,42], exhibited a high expression in hESCs in our dataset but was undetectable in later-stage populations. Similarly, miR-663, miR-1225-5p, and miR-638, previously reported as enriched in hESC-derived OLs [15], were not detected in any of the populations we analyzed. These discrepancies may reflect, as described above, differences in differentiation protocols or the platforms used to capture miRNAs compared to the previous studies. However, our OPC population, isolated based on OPC-specific PDGFRA expression, has been previously validated as closely resembling primary human OPCs [16], and the O4+ population has been confirmed to express mature OL markers. Moreover, NGS-based platforms capture miRNAs with higher sensitivity and lower false-positive rates compared to microarrays. Therefore, the miRNAs identified in our study are likely to more accurately reflect stage-specific expression in human OLLCs. Likewise, considering the enrichment of astrocyte markers in the non-OPC FT population [16], the miRNAs identified in this group are likely astrocyte-specific and may serve as potential markers for astroglia identity.

Our IPA-based pathway and target analysis of differentially expressed miRNAs, with particular focus on those enriched in OPCs and OLs, suggests that stage-specific miRNA expression patterns are associated with regulatory networks involving AKT and SMAD2/3 signaling pathways during OL lineage progression. The integration of these predicted pathways with additional signaling networks, including estrogen-receptor and insulin-signaling, highlights that a broader network of metabolic and extrinsic signals may influence OL lineage progression. Additionally, several predicted target genes identified through IPA, such as ZNF488, CSNK2B, KCNJ1, and DLX1, have previously been implicated in neural development and myelination. For example, ZNF488 (of Zfp488) and DLX1 are transcription factors that have been reported to cooperate with Olig2 to drive neural lineage specification and OL differentiation [53,56,69]. CSNK2B, which encodes the beta subunit of casein kinase 2, is a known critical regulator of cytoskeletal dynamics required for process extension during OL maturation [51,52]. Similarly, KCNJ1 (Kir7.1), a gene encoding the potassium channel, may modulate membrane potential and ion homeostasis, thereby influencing OL survival and functional maturation [54,55]. Together, these predicted miRNA–target relationships support the concept that miRNAs may influence OL development through coordinated regulation of multiple genes and signaling pathways, amplifying the impact of multiple small regulatory inputs to drive more precise, stage-specific outcomes in OL development. However, functional studies will be required to determine the direct regulatory roles of these miRNAs and their predicted targets in human OL lineage progression.

## 5. Conclusions

Our findings provide a comprehensive stage-specific miRNA expression profile and highlight novel candidate miRNAs potentially involved in human OPC and OL development. We validated the expression of selected candidate miRNAs in OPCs derived from multiple human ESC and iPSC lines using digital PCR, further supporting the robustness and reproducibility of our findings. However, this study is primarily exploratory by design. Functional validation of the identified miRNAs and their predicted targets is beyond the scope of the current work, which represents a limitation but also opens avenues for future studies. Future studies should include gain- and loss-of-function approaches to experimentally validate the functional roles of these miRNAs and their targets in oligodendrocyte biology, both in vitro and in relevant in vivo models. Such functional validation will be critical in informing whether these miRNAs and associated pathways represent potential therapeutic targets for remyelination therapy. If validated, these findings could ultimately contribute to novel drug development or aid in developing miRNA-based strategies aimed at restoring myelin integrity in demyelinating conditions.

## 6. Patents

X.C. is an inventor on an intellectual property disclosure related to the technology described in the manuscript that has been filed with Johns Hopkins University, which seeks to license the technology.

## Data Availability

The original contributions presented in this study are included in the article/Supplementary Material. All raw and processed next-generation sequencing (NGS) data generated in this study are available from the corresponding author, Xitiz Chamling (xchamli1@jhu.edu), upon reasonable request.

## Supplementary Materials

The following supporting information can be downloaded at: https://www.mdpi.com/article/10.3390/cells15151343/s1, Figure S1. Schematic overview of the approaches; Figure S2. Digital PCR data for selected miRNAs; Figure S3. Flow cytometry analysis of cell populations collected on Day 75; Figure S4. Ingenuity Pathway Analysis (IPA) of enriched miRNAs; Table S1. Quality control metrics for miRNA sequencing libraries; Table S2. Comprehensive annotation and processing metrics for miRNA sequencing data using miRge3.0 analysis tool; Table S3. Average of RPM values for previously known miRNAs at different stages of OL differentiation.

## Abbreviations

The following abbreviations are used in this manuscript:

CNS	Central nervous system
FACS	Fluorescence-activated cell sorting
FT	Flow-through
IPA	Ingenuity Pathway Analysis
MACS	Magnetic-activated cell sorting
NGS	Next-generation sequencing
NPC	Neural progenitor cell
OL	Oligodendrocyte
OPC	Oligodendrocyte progenitor cell
PCA	Principal Component Analysis
dPCR	Digital PCR
hESC	Human embryonic stem cell
hPSC	Human pluripotent stem cell
miRNA	MicroRNA
qPCR	Quantitative PCR
