# Supplementary material for: Profiling miRNAs Involved in Human Oligodendrocyte Precursor Cell Differentiation and Maturation
Source: Cells. 2026 Jul 27;15(15):1343. doi: 10.3390/cells15151343 (PMC13464101; doi:10.3390/cells15151343)
Supplement: Supplementary file 1 [file cells-15-01343-s001.zip › cells-4403317-supplementary.pdf]

Supplemental Information

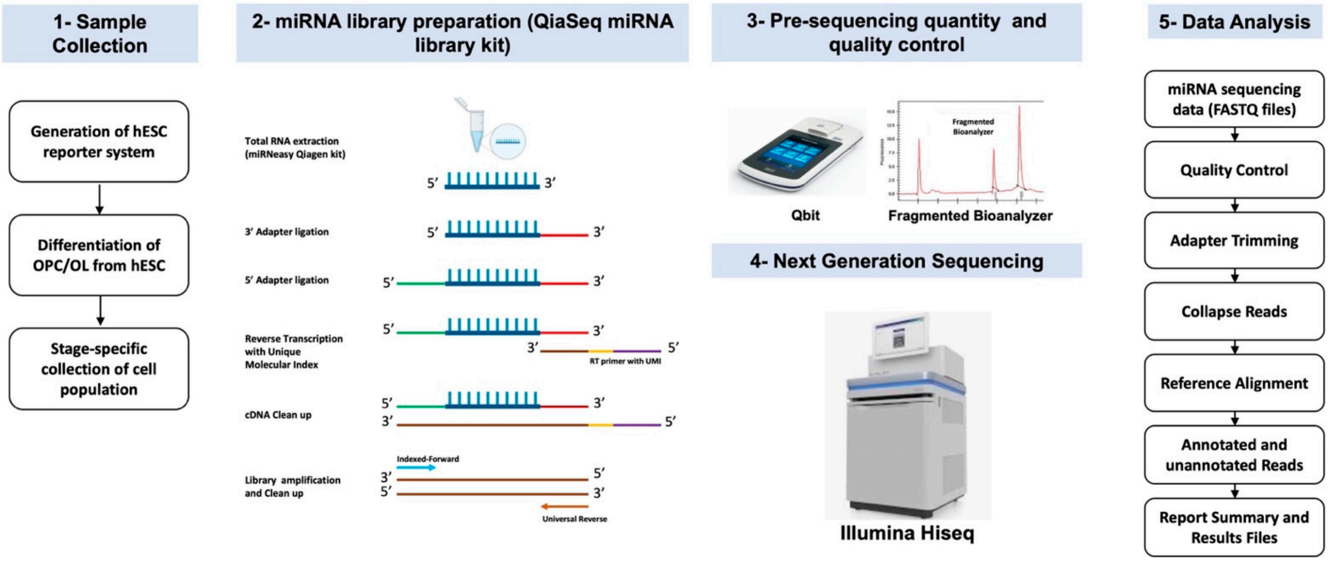

Figure S1. Schematic overview of the approaches. 1. Sample preparation and collection; **2.** miRNA library preparation; **3.** Quantity and quality control of the miRNA libraries using Q-bit and fragmented bioanalyzer, respectively; **4.** Next generation sequencing; and **5.** Data analysis pipeline.

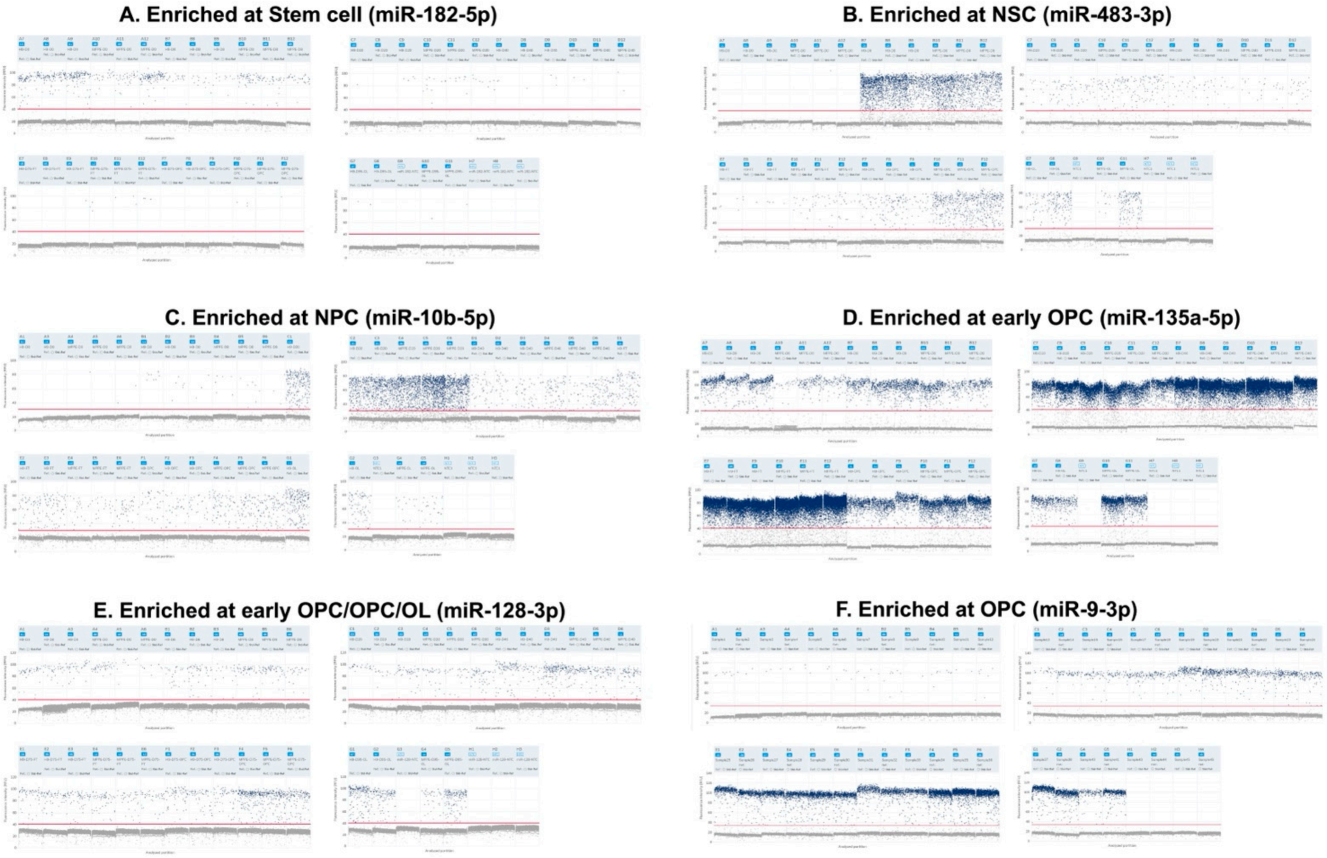

Figure S2. Digital PCR data for selected miRNAs. Each droplet in a sample is plotted on a graph of fluorescence intensity versus analyzed partitions. All droplets above the threshold intensity (indicated by the red line) are scored as positive, and those below the threshold are scored as negative.

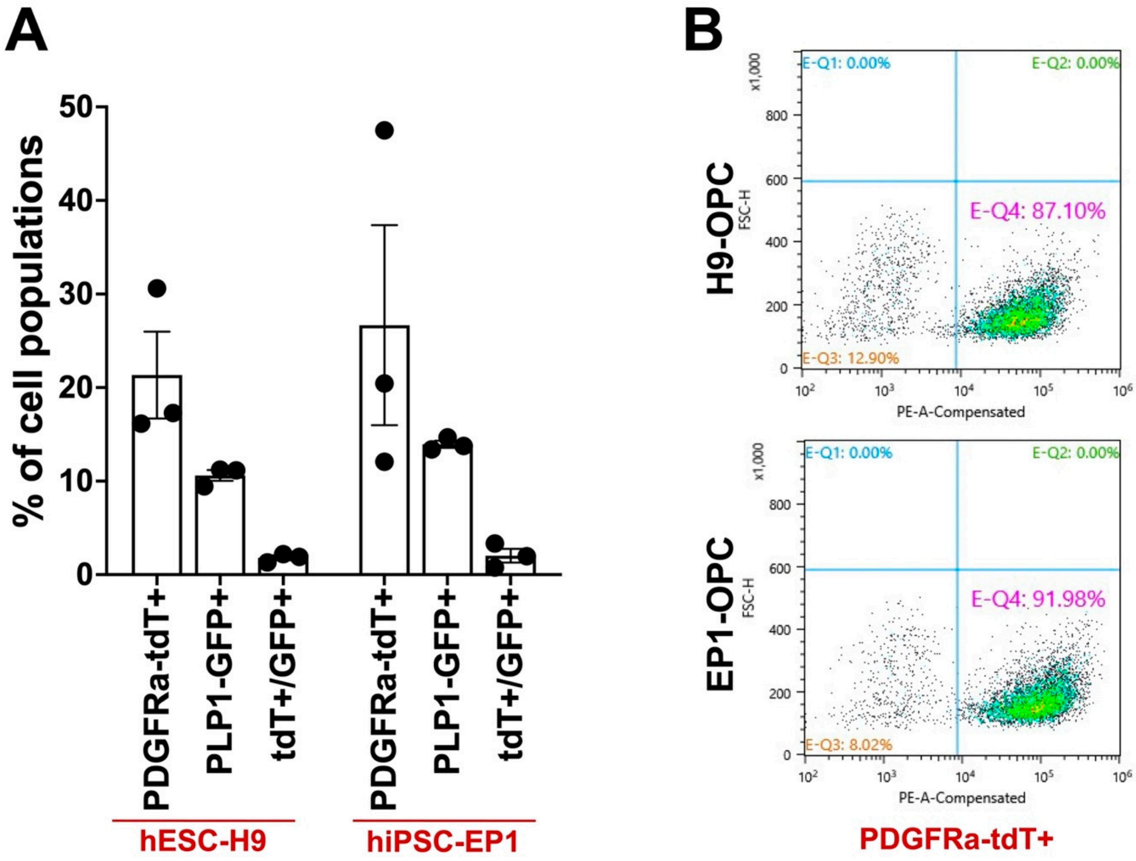

Figure S3. Flow cytometry analysis of cell populations collected on Day 75. (A) Comparison of PDGFRα-tdTomato+, PLP1-GFP+, and double-positive tdTomato+/GFP+ cell populations between hESC and hiPSC cell lines. (B) Flow cytometric assessment of OPC purity demonstrating differences between hESC-H9 (87.10%) and hiPSC-EP1 (91.98%) sorted populations.

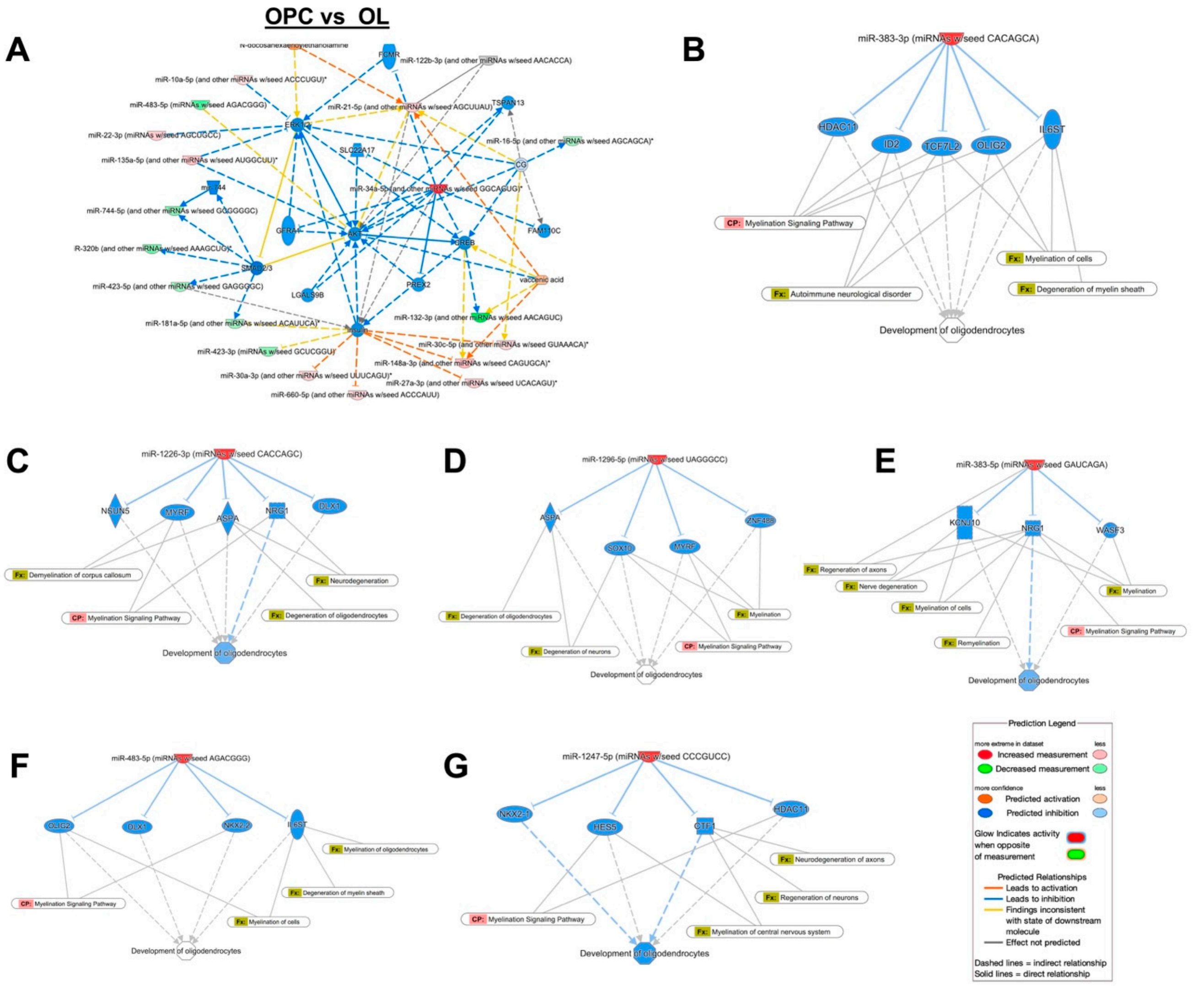

Figure S4. Ingenuity Pathway Analysis (IPA) of enriched miRNAs. **A)** IPA was performed on differentially expressed (DE) miRNAs identified in OL vs OPC comparison, which demonstrated predicted inhibition of ERK1/2, AKT, and SMAD2/3 signaling. B-G) Predicted mapped targets of OPC/OL-enriched miRNAs through which they potentially affect OL development and function: **B)** miR-383-5p, **C)** miR-1226-3p, **D)** miR-1296, **E)** miR-383-5p, **F)** miR-483-5p, and **G)** miR-1247-5p.

Table S1. Quality control metrics for miRNA sequencing libraries.

Sample Name	Reads	UMI Reads	Average Q score (UMI reads)	UMI Reads Annotated	UMI reads annotated with miRBase
A5_RPD18D0	14,974,048	2,520,433	41.5	1,078,328 (42.98%)	895,599 (35.70%)
B5_RPD19D0	16,442,729	1,571,528	42.26	601,167 (38.43%)	489,781 (31.31%)
G1_RPD16D0	14,614,708	1,249,154	40.24	408,227 (32.83%)	314,649 (25.30%)
A8_RPD19D8	19,988,989	3,324,287	43.58	1,476,086 (44.60%)	1,092,204 (33%)
C2_RPD16D8	13,519,390	886,790	40.06	69,857 (7.91%)	31,676 (3.59%)
C5_RDP18D8	14,430,153	2,382,437	41.81	938,077 (39.54%)	702,632 (29.62%)
B8_RPD19D20	16,213,768	5,803,958	41.25	4,618,787 (79.94%)	4,250,365 (73.57%)
C8_RPD16D20	15,680,864	5,508,300	41.49	4,434,803 (80.87%)	4,104,429 (74.85%)
D8_RPD18D20	18,054,393	6,912,916	41.73	5,559,181 (80.80%)	5,137,005 (74.66%)
E8_RPD16D40	20,746,728	8,774,574	42.52	7,486,984 (85.82%)	7,213,476 (82.69%)
F8_RPD18D40	18,715,408	8,253,066	41.42	6,893,479 (83.99%)	6,560,428 (79.94%)
G8_RPD19D40	22,534,316	5,976,405	43.3	4,492,290 (75.54%)	4,225,592 (71.06%)
B6_RPD19D75FT	15,386,516	2,062,445	40.35	912,777 (44.46%)	560,622 (27.31%)
G2_RPD16D75FT	16,402,448	4,276,176	40.68	2,675,657 (62.96%)	1,479,638 (34.81%)
H2_RPD18D75FT	14,578,280	1,856,463	40.35	684,823 (37.06%)	405,194 (21.93%)
A6_RPD19D75OPC	13,708,553	1,275,020	39.97	424,170 (33.42%)	312,642 (24.63%)
G4_RPD16D75OPC	14,314,670	1,259,380	40.33	424,510 (33.85%)	302,542 (24.13%)
H4_RPD18D75OPC	15,022,030	2,077,884	41.31	884,676 (42.77%)	696,301 (33.66%)
A7_RPD19D95OL	17,098,313	1,578,465	40.06	418,717 (26.64%)	274,652 (17.48%)
C6_RPD16D95OL	15,835,298	1,398,480	40.16	416,018 (29.88%)	266,342 (19.13%)
F6_RPD18D95OL	15,178,644	1,122,549	40.1	319,744 (28.61%)	223,049 (19.96%)

A table summarizing sequencing and quality control metrics for each miRNA library, including total reads, UMI-filtered reads, average Q scores for UMI reads, the number of UMI reads successfully annotated, and the subset of annotated UMI reads mapped to miRBase. These metrics were generated by Qiagen global analysis portal.

Table S2. Comprehensive annotation and processing metrics for miRNA sequencing data using miRge3.0 analysis tool.

Run	TimePoint	Unique miRNAs	Order	Total Input Reads	Trimmed Reads (all)	Trimmed Reads (unique)	All miRNA Reads	Filtered miRNA Reads	Unique miRNAs	Hairpin miRNAs	Mature tRNA Reads	Primary tRNA Reads	snoRNA Reads	rRNA Reads	ncRNA	mRNA Reads	Remaining Reads
A5_RPD18D0	D0	733	1	13368093	2920627	770168	884750	869412	733	11177	164561	94500	141294	438503	227172	24201	934469
B5_RPD19D0	D0	637	1	14681820	2212194	879712	485638	473155	637	4885	97314	53959	116781	313103	163579	15821	961114
G1_RPD16D0	D0	581	1	13058608	2589018	1435307	298589	291483	581	2097	103193	22218	131504	314509	153934	15336	1547638
A8_RPD19D8	D8	775	2	17871996	5063836	1917029	1106066	1092678	775	9694	320593	22482	219749	707238	541603	58434	2077977
C5_RDP18D8	D8	686	2	12902575	2970281	905672	679418	670142	686	3770	185262	16939	163928	559753	332924	35356	992931
B8_RPD19D20	D20	999	3	14496101	6680503	1069394	4289898	4280005	999	12356	341720	13625	237190	350362	336609	57052	1041691
C8_RPD16D20	D20	1017	3	14023077	6283770	931658	4183677	4173358	1017	13219	257183	25707	194340	308578	348933	55812	896321
D8_RPD18D20	D20	1039	3	16147113	8041790	1180471	5304615	5293235	1039	16679	386514	18760	278169	401894	413366	69284	1152509
E8_RPD16D40	D40	985	4	18560583	10172892	960799	8085498	8073420	985	11611	260240	72395	174680	336669	296795	36040	898964
F8_RPD18D40	D40	922	4	16736024	9179388	972725	7028225	7014891	922	12172	257033	66755	158903	385157	345932	44846	880365
G8_RPD19D40	D40	909	4	20147196	7236157	1398398	4565285	4548061	909	9511	254106	42537	121183	539044	298772	47876	1357843
B6_RPD19D75FT	D75-FT	458	5	13742857	2167157	495148	551051	542157	458	927	389157	870	63394	446307	170693	7700	537058
G2_RPD16D75FT	D75-FT	561	5	14660170	4952770	906135	1507636	1499714	561	2292	1394060	3169	117395	573007	248187	16129	1090895
H2_RPD18D75FT	D75-FT	419	5	13007799	1993051	523106	398801	389727	419	816	294263	831	65138	476804	179304	8396	568698
A6_RPD19D75OPC	D75-OPC	392	5	12233577	1337428	385694	305086	296979	392	865	123685	673	53956	274725	169272	6015	403151
G4_RPD16D75OPC	D75-OPC	414	5	12762309	1318084	364902	297042	288656	414	1014	152945	734	50720	272806	142842	5850	394131
H4_RPD18D75OPC	D75-OPC	479	5	13409594	2369227	621662	689760	679900	479	1746	297210	1509	90942	374670	240160	10684	662546
A7_RPD19D95OL	D95-OL	399	6	15260840	1554305	423494	274195	260058	399	977	113950	696	53402	442908	178107	7393	482677
C6_RPD16D95OL	D95-OL	391	6	14131662	1376357	362852	264420	254136	391	600	132723	603	34206	391768	111986	5980	434071
F6_RPD18D95OL	D95-OL	360	6	13540722	1131354	341572	221480	212067	360	509	84276	508	35934	271735	131705	4617	380590

A table summarizing read processing and annotation outputs for each sample, including total input reads, trimmed reads (all and unique), total miRNA reads, and filtered miRNA reads. It also reports the distribution of annotated RNA species, including unique miRNAs, hairpin miRNAs, mature tRNA reads, primary tRNA reads, snoRNA reads, rRNA reads, other non-coding RNAs, mRNA reads, and remaining unclassified reads. These metrics provide an overview of library composition and sequencing quality.

Table S3. Average of RPM values for previously known miRNAs at different stages of OL differentiation.

miRNA	D0	D8	D20	D40	D75-FT	D75-OPC	D95-OL
hsa-miR-100-3p	0.00	1.22	3.31	21.02	5.07	0.98	13.11
hsa-miR-100-5p	60.53	365.87	264.28	2098.72	1065.23	194.36	394.75
hsa-miR-103a-3p/107	24573.60	22295.87	37956.79	34670.41	9148.12	12966.08	11865.54
hsa-miR-103a-1-5p/103a-2-5p	6.69	0.61	0.89	0.86	0.00	0.00	0.00
hsa-miR-124-3p	94.66	207.72	324.56	2080.49	90.20	138.30	130.30
hsa-miR-124-5p	101.62	62.34	88.94	103.16	11.01	50.75	24.17
hsa-miR-125a-3p	56.44	111.85	23.72	35.87	67.17	125.38	90.42
hsa-miR-125a-5p	11050.26	23018.93	14257.48	48291.92	39810.06	43177.74	39041.32
hsa-miR-125b-2-3p	18.17	241.92	281.18	629.64	692.63	166.16	300.32
hsa-miR-125b-1-3p	37.76	232.50	35.99	712.45	1131.90	1222.37	824.01
hsa-miR-125b-5p	4845.99	48837.24	33835.94	170150.70	154063.89	126119.96	116180.63
hsa-miR-126-3p	1173.49	1522.85	3177.41	586.43	163.70	169.08	215.96
hsa-miR-126-5p	248.42	128.16	473.67	63.90	11.07	32.86	28.96
hsa-miR-130b-3p	1176.45	1844.52	2918.49	391.54	104.99	544.83	554.35
hsa-miR-130b-5p	365.46	1060.83	967.92	159.99	93.54	558.30	284.08
hsa-miR-130a-3p	5571.10	4326.97	6641.99	1522.43	473.95	911.22	948.53
hsa-miR-130a-5p	35.44	13.05	18.95	3.46	0.67	0.00	0.00
hsa-miR-138-1-3p	4.03	1.83	2.30	15.65	23.93	62.24	74.95
hsa-miR-138-2-3p	9.92	7.48	3.28	86.38	12.86	0.00	11.00
hsa-miR-138-5p	107.46	161.83	89.44	933.12	420.64	1026.71	1154.44
hsa-miR-142-3p	41.23	1.53	2.74	1.27	0.00	0.00	0.00
hsa-miR-142-5p	10.47	0.00	0.57	0.37	0.00	0.00	0.00
hsa-miR-145-3p	5.82	3.05	16.48	4.24	3.78	0.00	0.00
hsa-miR-145-5p	67.56	86.79	173.37	42.50	120.83	50.74	99.69
hsa-miR-146a-3p	0.00	0.00	0.00	0.12	0.00	0.00	0.00
hsa-miR-146a-5p	352.13	39.29	47.11	38.91	42.33	18.25	30.05
hsa-miR-146b-3p				4.56	19.32	5.39	8.97
hsa-miR-146b-5p				318.17	219.26	105.30	109.41
hsa-miR-152-3p	60.72	312.80	625.30	390.57	340.52	101.91	282.12
hsa-miR-152-5p	0.00	0.92	1.42	1.54	0.00	0.00	0.00
hsa-miR-155-5p	920.61	264.63	142.09	21.78	60.88	78.30	52.16
hsa-miR-155-3p	0.00	0.00	0.00		0.00	0.00	0.00
hsa-miR-16-1-3p	0.00	4.24	6.48	2.85	0.44	17.59	0.00
hsa-miR-16-2-3p					2.56	9.87	10.22
hsa-miR-16-5p	35087.28	48237.27	139992.19	54091.38	20723.16	32775.12	40444.01
hsa-miR-17-3p	342.93	118.57	378.83	147.96	25.64	100.36	58.93
hsa-miR-181a-3p	26.88	52.50	98.02	649.42	211.31	593.59	617.89
hsa-miR-181a-5p	218.43	965.25	493.43	5949.51	10684.96	23551.40	14647.34
hsa-miR-181b-3p	0.00	0.61	4.27	14.39	0.00	29.29	11.63
hsa-miR-181b-5p	102.41	433.96	573.72	1685.69	2362.08	5139.21	3775.47
hsa-miR-184	197.14	1046.22	149.21	15.80	18.67	16.13	30.23
hsa-miR-191-3p	170.69	146.89	107.55	40.33	22.76	37.10	28.67
hsa-miR-191-5p	11173.87	14901.74	13299.92	7939.10	3601.57	4973.84	5047.67
hsa-miR-199a-3p/199b-3p	187.58	430.15	784.80	221.43	1343.87	1029.29	465.94
hsa-miR-199a-5p	0.00	4.81	11.86	10.35	50.01	34.64	17.05
hsa-miR-19b-3p	7297.26	2708.75	6955.29	937.48	343.87	1184.55	1381.41
hsa-miR-19b-2-5p	4.67	1.53	2.08	0.00	0.00	0.00	0.00
hsa-miR-19b-1-5p	19.97	6.87	25.29	6.71	0.00	0.98	2.62
hsa-miR-200a-3p	533.54	93.73	60.36	15.87	13.51	13.35	16.89
hsa-miR-200a-5p	30.69	0.00	1.09	0.00	0.00	0.00	0.00
hsa-miR-204-3p	16.46	83.49	5.04	1.07	0.00	0.00	0.00
hsa-miR-204-5p	1827.44	1734.64	695.98	238.22	81.59	27.66	42.32
hsa-miR-20a-3p	115.11	39.02	160.82	38.32	8.77	27.15	22.02
hsa-miR-20a-5p	11587.41	7284.94	23509.87	3106.72	893.18	2365.63	2499.70
hsa-miR-21-3p	129.65	212.66	70.90	43.28	352.41	152.61	198.81
hsa-miR-21-5p	7852.44	12633.62	45074.97	20224.18	46424.12	11592.82	30477.40
hsa-miR-212-3p	0.00	3.36	2.76	12.21	1.33	3.92	0.00
hsa-miR-212-5p	0.00	6.84	5.35	34.34	11.95	21.57	12.57
hsa-miR-214-3p	0.00	3.21	4.76	1.78	55.79	36.95	10.49
hsa-miR-214-5p	0.00	0.00	0.19	0.45	4.00	0.00	0.00
hsa-miR-219b-3p	0.00	0.00	2.70	0.81	0.00	0.00	0.00
hsa-miR-219b-5p	0.00	0.00	61.85	27.20	0.00	0.00	9.18
hsa-miR-219a-1-3p	22.01	865.34	599.29	130.25	51.47	92.45	53.61
hsa-miR-219a-2-3p	781.58	38761.24	38235.86	2773.89	979.13	5535.14	9354.68
hsa-miR-219a-5p	159.38	2513.65	8714.87	727.46	229.53	759.78	474.81
hsa-miR-23a-3p/23b-3p	705.36	1995.87	2141.32	6282.26	4952.05	1427.05	2455.79
hsa-miR-23b-5p	27.82	153.29	14.60		90.42	28.04	54.22
hsa-miR-25-5p	1313.72	1111.61	148.51	50.71	62.67	340.26	214.97
hsa-miR-25-3p				5824.26	2375.98	5133.72	4953.33
hsa-miR-26a-5p	6399.46	18621.68	36758.49	32727.17	11422.19	10787.81	12436.88
hsa-miR-3065-3p	37.94	32.50	122.04	153.86	93.84	29.89	143.18

miRNA	D0	D8	D20	D40	D75-FT	D75-OPC	D95-OL
hsa-miR-3065-3p	37.94	32.50	122.04	153.86	93.84	29.89	143.18
hsa-miR-3065-5p	147.82	235.91	361.23	432.43	367.03	112.05	510.00
hsa-miR-30a-3p	113.89	2251.82	2411.10	920.71	1148.58	246.72	586.99
hsa-miR-30a-5p	743.21	14624.07	17948.88	8117.06	8940.71	3160.95	6792.39
hsa-miR-30b-3p	12.20	17.88	5.28	3.40	6.59	12.71	2.62
hsa-miR-30b-5p/30c-5p	1781.03	4842.71	6320.90	2603.94	1691.28	1396.33	1904.90
hsa-miR-31-3p	7.08	1.83	4.72	1.63	0.00	0.00	3.93
hsa-miR-31-5p	586.26	582.91	504.76	134.28	167.11	35.68	94.20
hsa-miR-335-3p	509.84	487.74	1405.23	276.58	92.02	89.19	173.91
hsa-miR-335-5p	1173.60	1483.81	3198.81	1914.87	782.76	1118.60	816.15
hsa-miR-339-3p	112.38	318.90	341.21	327.94	120.82	174.39	111.58
hsa-miR-339-5p	914.28	2683.42	2171.62	241.25	209.92	381.58	373.38
hsa-miR-338-3p	48.24	89.33	151.99	510.36	64.71	342.89	573.03
hsa-miR-338-5p	1.15	0.61	1.67	2.26	0.00	0.00	0.00
hsa-miR-34a-3p	6.47	6.11	22.07	5.57	4.85	0.00	5.13
hsa-miR-34a-5p	2556.30	10318.66	16654.89	3665.11	4420.99	3169.51	3867.23
hsa-miR-34b-3p	12.26	15.97	17.46	6.54	12.34	0.00	12.85
hsa-miR-34b-5p	39.96	8.06	13.69	9.79	3.79	0.00	20.80
hsa-miR-34c-3p	4.23	11.88	10.63	6.12	14.77	0.00	5.13
hsa-miR-34c-5p	32.58	35.68	105.75	60.48	68.00	5.25	129.79
hsa-miR-657	0.00	0.00	0.00	0.24	0.00	0.00	0.00
hsa-miR-665-3p	0.00	0.00	0.87	0.00	0.00	0.00	0.00
hsa-miR-7-5p	8810.08	3358.92	11788.78	20200.74	1665.16	2134.97	1901.45
hsa-miR-7-2-3p	0.00	0.00	3.92	22.84	7.01	11.64	5.25
hsa-miR-7-1-3p	42.05	8.67	36.72	24.38	4.65	5.77	3.14
hsa-miR-9-3p	256.09	1135.91	852.46	6708.54	14461.82	22846.07	18451.32
hsa-miR-9-5p	3347.93	16446.69	16925.51	129049.85	101366.05	121344.87	113374.99
hsa-miR-92a-1-5p	135.40	97.01	8.25	2.85	0.44	11.84	9.55
hsa-miR-92a-2-5p	151.89	147.29	6.37	0.00	3.57	2.45	3.14
hsa-miR-92a-3p	150501.61	136981.37	43495.65	7697.96	10160.28	21238.37	18104.33
hsa-miR-92b-3p	10142.88	17756.81	5367.59	3246.43	15666.15	3068.27	4545.30
hsa-miR-92b-5p	1383.08	4639.50	497.58	278.29	2481.11	718.76	1002.48
hsa-miR-99a-3p	21.06	251.49	219.16	411.09	348.27	108.50	194.61
hsa-miR-99a-5p	198.67	2038.12	4904.28	6321.06	4697.48	946.83	2223.12
hsa-miR-99b-3p	429.99	684.10	542.34	549.27	456.86	751.18	558.09
hsa-miR-99b-5p	3249.58	6406.89	6986.68	11349.51	4613.94	5813.12	4913.75
hsa-miR-130a-3p	5571.10	4326.97	6641.99	1522.43	473.95	911.22	948.53
hsa-miR-130a-5p	35.44	13.05	18.95	3.46	0.67	0.00	0.00
hsa-miR-130b-5p	365.46	1060.83	967.92	159.99	93.54	558.30	284.08
hsa-miR-130b-3p	1176.45	1844.52	2918.49	391.54	104.99	544.83	554.35
hsa-miR-15b-3p	210.51	180.19	414.90	64.57	15.03	48.72	66.44
hsa-miR-15b-5p	2900.90	3109.74	2944.79	861.08	709.21	1632.43	998.74
hsa-miR-18a-3p	593.74	304.81	136.91	16.96	2.89	28.13	24.50
hsa-miR-18a-5p	826.67	542.68	2117.90	118.14	56.50	137.84	193.91
hsa-miR-199b-5p	60.11	80.99	235.01	21.46	42.89	105.18	100.73
hsa-miR-27a-3p/27b-3p	1052.62	3337.67	5796.05	12687.59	6630.45	1669.50	3787.23
hsa-miR-27a-5p	0.00	40.30	26.65	29.75	39.73	4.49	19.53
hsa-miR-27b-5p	10.70	2.14	9.68	24.45	22.22	0.00	12.82
hsa-miR-363-3p	3491.95	4507.84	8685.98	414.49	215.74	414.47	424.93
hsa-miR-363-5p	133.28	69.21	15.53	3.10	0.00	17.19	0.00
hsa-miR-424-3p	11.07	171.10	14.44	5.23	26.65	96.43	51.61
hsa-miR-424-5p	24.27	65.09	207.32	94.98	100.61	298.01	314.32
hsa-miR-4443	0.00	29.90	14.12	3.82	96.95	151.20	88.79
hsa-miR-450a-1-3p	0.00	10.32	11.13	8.29	7.54	25.26	9.43
hsa-miR-450a-2-3p	4.41	9.89	5.38	4.93	1.33	15.42	3.85
hsa-miR-450a-5p	1.41	2.52	11.95	6.86	0.89	1.47	9.84
hsa-miR-450b-5p	24.68	110.70	257.66	88.60	39.25	127.06	148.49
hsa-miR-503-3p	7.26	162.86	12.99	6.72	13.19	94.98	62.63
hsa-miR-503-5p	89.05	553.22	222.96	82.70	157.07	779.40	479.33
hsa-miR-542-3p	30.17	115.72	311.59	158.22	128.86	358.74	377.75
hsa-miR-542-5p	8.79	36.53	50.82	49.53	38.48	153.09	94.89
hsa-miR-582-3p	1.92	3.36	4.09	27.30	13.88	18.37	34.57
hsa-miR-582-5p	0.00	0.00	1.64	5.49	1.33	5.43	5.25
hsa-let-7a-3p	0.00	0.00	0.38	14.36	16.12	26.44	41.93
hsa-let-7a-5p/7c-5p	4826.37	9690.18	779.49	71243.40	202829.96	109196.53	116802.95
hsa-let-7b-3p	0.00	0.00	0.00	0.37	0.00	37.85	42.93
hsa-let-7b-5p	809.39	3554.35	90.86	1736.65	126524.15	189549.13	199549.52
hsa-let-7c-3p	0.00	0.00	0.48	84.59	37.36	24.00	30.70
hsa-let-7a-5p/7c-5p	4826.37	9690.18	779.49	71243.40	202829.96	109196.53	116802.95
hsa-let-7d-3p	18.13	1.99	0.16	15.32	128.87	284.03	232.39
hsa-let-7d-5p	55.60	106.15	7.32	631.03	1357.46	2317.20	1659.87
